# Good News about Bad News: Gamified Inoculation Boosts Confidence and Cognitive Immunity Against Fake News

**DOI:** 10.5334/joc.91

**Published:** 2020-01-10

**Authors:** Melisa Basol, Jon Roozenbeek, Sander van der Linden

**Affiliations:** 1 Department of Psychology, University of Cambridge, UK

**Keywords:** Judgment, Decision making: Reasoning

## Abstract

Recent research has explored the possibility of building attitudinal resistance against online misinformation through psychological inoculation. The inoculation metaphor relies on a medical analogy: by pre-emptively exposing people to weakened doses of misinformation cognitive immunity can be conferred. A recent example is the *Bad News* game, an online fake news game in which players learn about six common misinformation techniques. We present a replication and extension into the effectiveness of *Bad News* as an anti-misinformation intervention. We address three shortcomings identified in the original study: the lack of a control group, the relatively low number of test items, and the absence of attitudinal certainty measurements. Using a 2 (treatment vs. control) × 2 (pre vs. post) mixed design (N = 196) we measure participants’ ability to spot misinformation techniques in 18 fake headlines before and after playing *Bad News*. We find that playing *Bad News* significantly improves people’s ability to spot misinformation techniques compared to a gamified control group, and crucially, also increases people’s level of confidence in their own judgments. Importantly, this confidence boost only occurred for those who updated their reliability assessments in the correct direction. This study offers further evidence for the effectiveness of psychological inoculation against not only specific instances of fake news, but the very strategies used in its production. Implications are discussed for inoculation theory and cognitive science research on fake news.

## Introduction

The prevalence and propagation of online misinformation is a threat to science, society, and democracy (Lazer et al., 2018; Lewandowsky et al., 2017; van der Linden, Maibach, et al., 2017). Recent research has shown that increased exposure to false and misleading information can have serious consequences ranging from societal misconceptions around climate change and vaccinations (Schmid & Betsch, 2019; van der Linden, Leiserowitz, et al., 2017) to physical danger and death (Arun, 2019). Although much research continues to debate the effectiveness of debunking and fact-checking (Chan et al., 2017; Nyhan & Reifler, 2019), a large body of research in cognitive psychology emphasises the continued influence of misinformation: falsehoods are difficult to correct once they have manifested themselves in memory (Lewandowsky et al., 2012) and repeated exposure increases the perceived accuracy of fake news (Pennycook et al., 2018). Consequently, some scholars have started to explore the possibility of “*prebunking*”, i.e. preventative strategies against the spread of misinformation (Roozenbeek & van der Linden, 2018, 2019). Because the spread of fake news in online networks bears close resemblance to the manner in which a virus replicates (Kucharski, 2016), one promising avenue has been the revival of inoculation theory.

Cognitive inoculation is based on the biological analogy of vaccine immunisation (McGuire & Papageorgis, 1961; McGuire, 1964). It posits that the process of injecting a weakened dose of a virus to activate antibody production (to help confer resistance against future infection) can similarly be applied to the context of information processing. In other words, by warning and exposing people to severely weakened doses of attitudinal challenges, cognitive resistance or “mental antibodies” are generated against future persuasion attempts (Compton & Pfau, 2005), partly by fortifying the structure of associative memory networks (Pfau et al., 2005). Although meta-analyses have shown that inoculation messages are effective (Banas & Rains, 2010), early inoculation research was mostly restricted to “cultural truisms”, i.e. beliefs so commonly shared across the social milieu that the notion of persuasive attacks against them appeared unlikely (McGuire, 1964). In the real-world, however, people will often hold very different prior beliefs about a particular issue. Accordingly, McGuire’s restrictive use of the metaphor has been criticized (Pryor & Steinfatt, 1978) and ultimately led to a rethinking of the medical analogy (Wood, 2007). In fact, more recent studies have demonstrated the efficacy of inoculation even when participants have differing prior attitudes, for example in the context of disinformation campaigns about climate change (Cook et al., 2017; van der Linden, Leiserowitz, et al., 2017). Accordingly, the consensus view is that “the analogy is more *instructive* than restrictive” (Compton, 2013, p. 233). Of course, from a theoretical point of view, we cannot speak of purely prophylactic inoculation in the context of most real-world settings but just as medicine has advanced to distinguish between *prophylactic* and *therapeutic* vaccines, therapeutic inoculation approaches can still confer protective benefits even among those already “afflicted” by boosting immune responses in the desired direction (Compton, 2019). Yet, it remains unclear whether the same theoretical mechanisms that facilitate prophylactic inoculation (e.g. confidence in defending one’s beliefs) also boost the efficacy of therapeutic inoculation.

**Figure 1:**
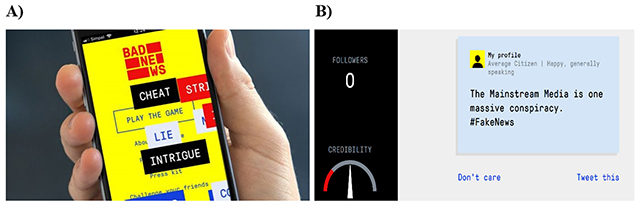
Landing screen *Bad News* (**Panel A**) and simulated twitter engine (**Panel B**).

Moreover, current inoculation research suffers from two primary limitations; 1) scholarship has predominantly focused on conferring attitudinal resistance against specific issues and 2) preemptive refutation has traditionally been done in a passive rather active manner (Banas & Rains, 2010). These two issues substantially limit both the scalability and generalisability of the “vaccine” metaphor (Bonetto et al., 2018; Roozenbeek & van der Linden, 2019). Accordingly, recent research has focused on the possibility of a “broad-spectrum vaccine” against misinformation (Roozenbeek & van der Linden, 2018, 2019). The broad-spectrum approach requires two theoretical innovations; 1) shifting focus away from pre-emptively exposing participants to weakened examples of specific instances of (mis)information to pre-emptively exposing participants to weakened examples of the *techniques* that underlie the production of most misinformation and 2) revisiting McGuire’s original prediction (McGuire & Papageorgis, 1961) that active inoculation (letting participants generate their own “antibodies”) would be more effective in conferring resistance to persuasion than when participants are provided with a defensive pre-treatment in a passive manner. In a novel paradigm pioneered by Roozenbeek and van der Linden (2019), participants enter a simulated social media environment (Twitter) where they are gradually exposed to weakened “doses” of misinformation strategies and actively encouraged to generate their own content. The intervention is a free social impact game called *Bad News* (www.getbadnews.com; Figure 1A), developed in collaboration with the Dutch media platform DROG (DROG, 2018), in which players learn about six common misinformation techniques (impersonating people online, using emotional language, group polarisation, spreading conspiracy theories, discrediting opponents, and trolling, Figure 1B).

The purpose of the game is to produce and disseminate disinformation in a controlled environment whilst gaining an online following and maintaining credibility. Players start out as an anonymous netizen and eventually rise to manage their own fake news empire. The theoretical motivation for the inclusion of these six strategies are explained in detail in Roozenbeek and van der Linden (2019) and cover many common disinformation scenarios including false amplification and echo chambers. Moreover, although the game scenarios themselves are fictional they are modelled after real-world events. In short, the gamified inoculation treatment incorporates an active and experiential component to resistance-building.

The initial study by Roozenbeek and van der Linden (2019) relied on a self-selected online sample of approximately 15,000 participants in a pre-post (within) gameplay design. Although the study provided preliminary evidence that the game increases people’s ability to detect and resist a whole range of misinformation (in the form of deceptive Twitter posts), the study suffered from a number of important theoretical and methodological limitations. For example, although the original study did include various “real news” control items, it lacked a proper randomized control group. This is important because there could be a secular trend so that people downgrade their reliability ratings of the fake tweets (pre-post) regardless of what intervention they are assigned to. Second, because the testing happened within the game environment, the original study only included a limited number of fake news items (one survey item per misinformation technique). Third, on a theoretical level, the study only looked at reliability judgments and thus could not determine how confident or certain people actually were in their beliefs. This is important, because attitude certainty (a dimension of attitude strength) is generally regarded as the conviction that held attitudes are correct (Tormala & Petty, 2004) and functions as a critical mechanism in resisting persuasion attempts (Compton & Pfau, 2005). Accordingly, this study addresses three key shortcomings in the original research by 1) including a randomized control group, 2) adding a larger battery of items, and 3) evaluating whether the intervention also boosts confidence in reliability judgments.

## Participants and procedure

This study employed a 2 (*Bad News*. vs. Control) * 2 (pre-post) mixed design to test the efficacy of active (gamified) inoculation in conferring attitudinal resistance to misinformation. The independent variable consisted of either the treatment condition in which participants played the *Bad News* game or a control condition in which participants were assigned to play *Tetris* (to control for gamification; *Tetris* specifically was chosen because it is in the public domain and requires little prior explanation before playing).

Following Roozenbeek and van der Linden (2019), the dependent variable consisted of an assessment of the reliability of 18 misinformation headlines in the form of Twitter posts (please see Supplementary Figure S5). As the *Bad News* game covers six misinformation techniques, three items per technique were included.^1^ These Twitter posts were created to be realistic, but not real, both to avoid memory confounds (participants may have seen “real” fake news headlines before) and to able to experimentally isolate the misinformation techniques. Taking into account the average inoculation effect reported in previous research (Roozenbeek & van der Linden, 2019), an a priori power analysis was conducted with G* power using α = 0.05, *f* = 0.26 (*d* = 0.52) and power of 0.90 with two experimental conditions. The minimal sample size required for detecting the main effect was approximately 158. A total of 197 participants were recruited through the online crowdsourcing platform, *Prolific Academic*, which has been reported to produce higher data quality than *MTurk* (Peer et al., 2017). Consenting participants (58% male, modal age bracket = 18–24, 20% higher educated, 61% liberal, 80% white^2^) completed the survey, were debriefed, and paid £2.08 in compensation. This study was approved by the Cambridge Psychology Research Ethics Committee.

A plug-in was created so that the game could be embedded in Qualtrics and pre-post testing could take place outside of the game environment to further enhance ecological validity. Upon giving informed consent, participants were randomly presented with 18 fictitious Twitter posts (Figure S5) and on a standard 7-point scale, reported on how reliable they received each post to be and how confident they were in their judgements. Subsequently, participants were randomly assigned to a condition. In the inoculation condition participants (*n* = 96) were asked to play the “*Bad News*” game for about 15 minutes. Participants were assigned a password for completion which they could only receive after completing the final level (badge). Participants (*n* = 102) in the control condition played *Tetris* for 15 minutes in the same manner. After treatment exposure, all participants were asked to complete the same set of outcome measures.

## Outcome Measures

### Perceived reliability

To assess participants’ perceived reliability, a single-item measure was presented alongside 18 (6*3) fake Twitter posts (example item polarization; “*New study shows that right-wing people lie more often than left-wing people”*, see Figure S5). Participants reported the perceived reliability of each post on a 7-point Likert-scale from not reliable at all (1), neutral (4) to very reliable (7). Following Roozenbeek and van der Linden (2019), to form a general fake news scale of perceived reliability, all 18 fake news items were averaged. An initial reliability analysis suggested good internal consistency (*M* = 3.17, *SD* = 0.85, α = 0.84) of the 18-item fake news scale. A subsequent exploratory principal component analysis (PCA) was also run on the fake news items. According to the Kaiser criterion, results indicated that the items clearly loaded on a single dimension with an eigenvalue of 3.15, accounting for 53% of the variance (please see Scree plot, Supplementary Figure S6). Thus, for ease of interpretation and to limit multiple testing, all 18 items were collapsed and treated as one overall measure of fake news judgments. Nonetheless, descriptive statistics for badge-level results are also presented in Supplementary Table 1.

### Attitudinal certainty

Similarly, a single-item measure was presented alongside each of the news items, asking participants to indicate how confident they are in their reliability assessment on a 7-point Likert scale, ranging from not at all confident (1) to neutral (4) to very confident (7). Scale reliability analysis on the averaged 18 attitude certainty items (6*3) indicated high internal validity (*M* = 5.23, SD = 0.84, α = .89). Similarly, PCA results indicated that the items loaded on a single dimension with an eigenvalue of 3.88, accounting for 65% of variance (Supplementary Figure S7, for badge-level results see Table S2).

### Political ideology

Political ideology was measured on a standard self-placement scale, ranging from 1 = very conservative, 4 = moderate, to 7 = very liberal. Although often more diverse than Mturk (Peer et al., 2017), the Prolific sample (*M* = 4.69, SD = 1.42) was fairly liberal with 21% conservatives, 18% moderates, and 61% identifying as liberal.

## Results

**Figure 2:**
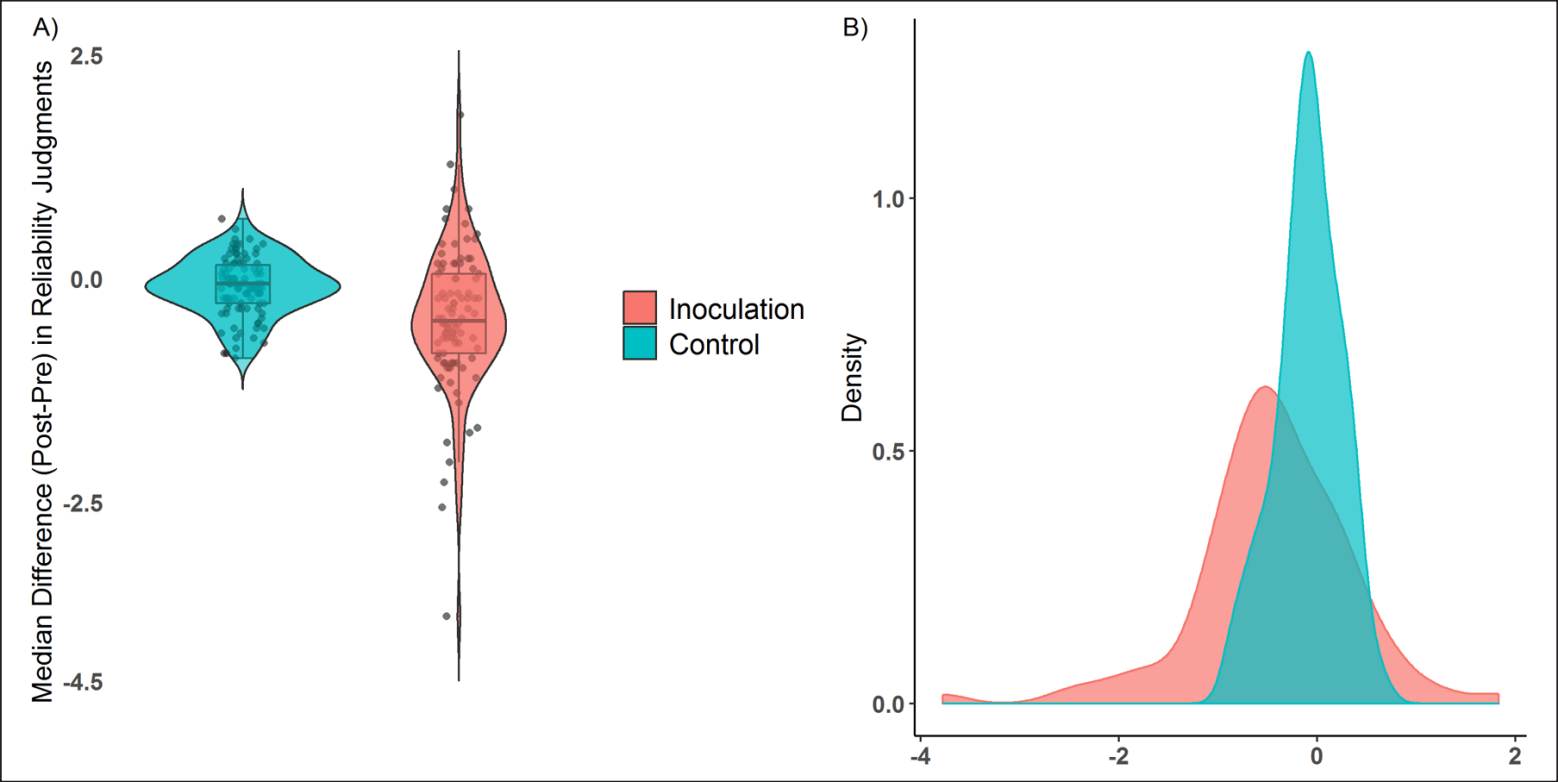
Median difference (post-pre) in reliability assessments of fake news items across treatment conditions with jitter (**Panel A**) and density plots of the data distributions (**Panel B**).

A One-way ANOVA was conducted to compare the effect of treatment condition (inoculation, control) on the difference in pre-and-post reliability scores of the fake news items. Results demonstrate a significant main effect of treatment condition on aggregated reliability judgements: *F*(1, 196) = 17.54, MSE = 0.36, *p* < .001, η^2^ = .082).^3^ Specifically, compared to the control condition, the shift in post-pre difference scores was significantly more negative in the inoculation condition (*M* = –0.09 vs *M* = –0.45, *M*_diff_ = –0.36, 95% CI [–0.19, –0.52], *d* = –0.60, Figure 2). A separate two-way ANOVA revealed no main effect *F*(2, 179) = 2.80, *p* = 0.06 nor interaction *F*(2, 179) = 0.96, *p* = 0.38 with political ideology.^4^ In short, compared to their assessments on the pre-test, individuals demonstrated a larger decrease in perceived reliability of fake news items when in the inoculation group versus the control condition. Similar patterns were observed at the badge level in the game (please see Supplementary Table 1) although there was some heterogeneity across badges with average effect-sizes ranging from *d* = 0.14 (polarization) to *d* = 0.58 (discrediting).

**Figure 3:**
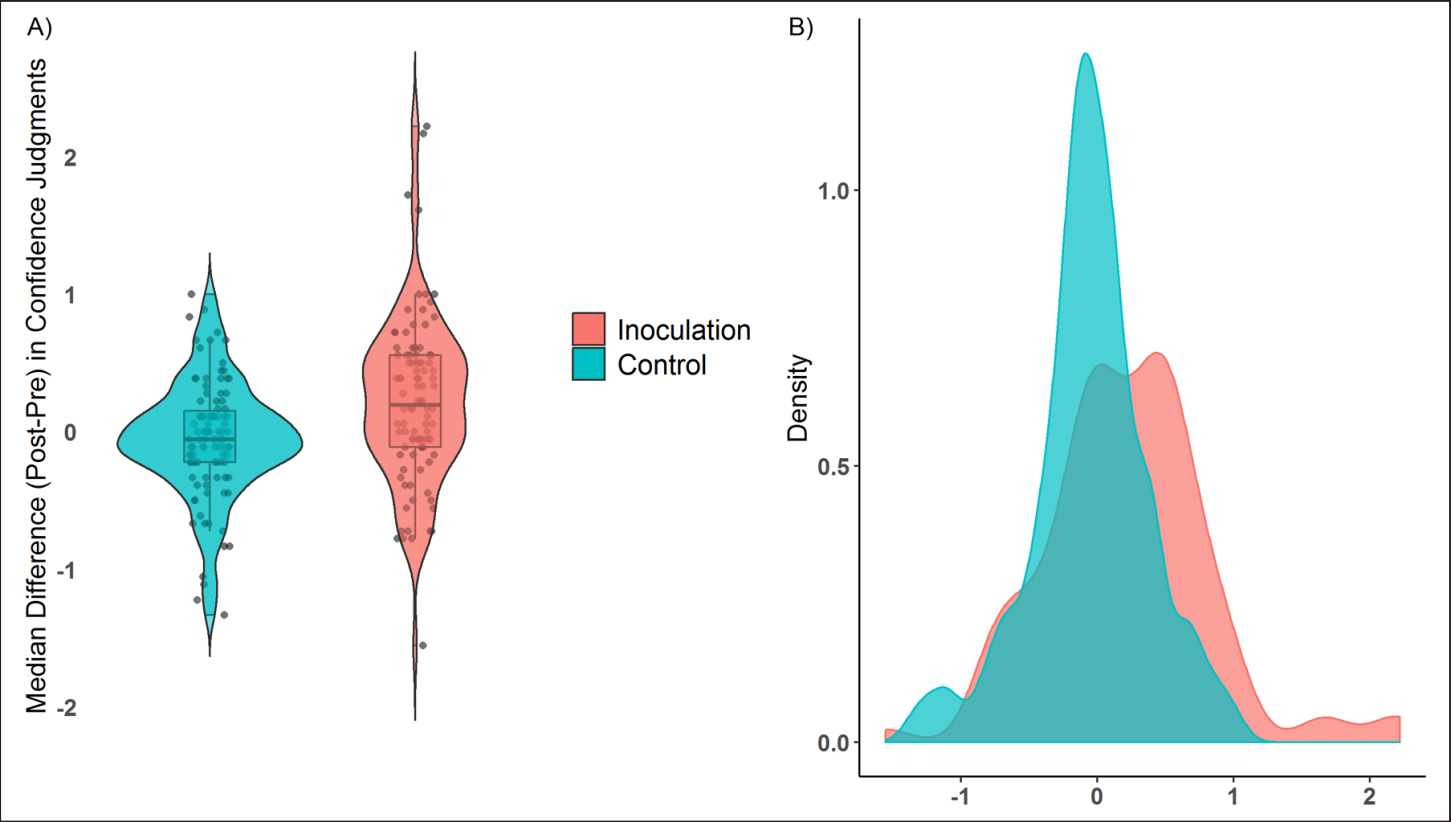
Median change scores (post-pre) of confidence in reliability judgments across treatment conditions with jitter (**Panel A**) and density plots of the data distributions (**Panel B**).

Furthermore, a one-way ANOVA also demonstrated a significant main effect of treatment condition on (post-pre) confidence scores (Figure 3), *F*(1, 196) = 13.49, MSE = 0.27, *p* < .001, η^2^ = .06. Mean difference comparisons across conditions indicate a significantly higher (positive) difference score in the inoculation group compared to the control condition (*M* = 0.22 vs. *M* = –0.06, *M*_diff_ = 0.27, 95% CI [0.13, 0.42], *d* = 0.52).^5^ This suggests that compared to their assessments prior to treatment exposure, individuals demonstrated a larger increase in confidence in the inoculation versus the control condition. Once again a two-way ANOVA revealed no main effect *F*(2, 179) = 1.22, *p* = 0.30 nor interaction *F*(2, 179) = 0.14, *p* = 0.87 with political ideology. At the badge level (Supplementary Table 2), effect-sizes for increased confidence ranged from *d* = 0.23 (discrediting) to emotion (*d* = 0.49). Importantly, the increase in confidence only occurred for those (71%) who broadly updated their reliability judgments in the right direction^6^ (*M_inoculation_* = 0.29 vs. *M_control_* = –0.02 *M*_diff_ = 0.31, 95%[0.13, 0.49], *t*(126) = 3.37, *p* < 0.01). In contrast, no gain in confidence was found among those who either did not change or updated their judgments in the wrong direction (*M_inoculation_* = 0.03 vs. *M_control_* = –0.11, *M*_diff_ = 0.14 95%[–0.11, 0.39], *t*(68) = 1.13, *p* = 0.26).

## Discussion and conclusion

This study successfully demonstrated the efficacy of a “broad-spectrum” inoculation against misinformation in the form of an online fake news game. Using a randomized design, multiple items, and measures of attitudinal certainty, we expand on the initial study by Roozenbeek and van der Linden (2019). Overall, we find clear evidence in support of the intervention. Whereas Roozenbeek and van der Linden (2019) reported an average effect-size of *d* = 0.52 for aggregated reliability judgments using a self-selected within-subject design, we find very similar effect-sizes in a randomized controlled design (*d* = 0.60). The range in effect-sizes observed on the badge level (*d* = 0.14 to *d* = 0.58) are also similar to what Roozenbeek and van der Linden (2019) reported (*d* = 0.16 to *d* = 0.36), and can be considered sizeable in the context of resistance to persuasion research (Banas & Rains, 2010; Walter & Murphy, 2018). In fact, Funder and Ozer (2019) recommend describing these effects as medium to large and practically meaningful, especially considering the refutational-*different* rather than refutational-same approach adopted here, i.e. in the game, participants were trained on *different* misleading headlines than they were tested on pre-and-post. Moreover, the fictitious nature of the items help rule out potential memory confounds and the lack of variation on the measures (pre-post) in the control group should decrease concerns about potential demand characteristics.

Importantly, consistent with Roozenbeek and van der Linden (2019), none of the main effects revealed an interaction with political ideology, suggesting that the intervention works as a “broad-spectrum” vaccine across the political spectrum. However, it is interesting that in both studies, the smallest effect is observed for the polarization badge. One potential explanation for the lower effect on polarization is confirmation bias: in the game, decisions can still be branched in an ideologically congenial manner. Given the worldview backfire effect (Lewandowsky et al., 2012), future research should evaluate to what extent inoculation is effective for ideologically congruent versus non-congruent fake news. Nonetheless, these results complement prior findings which suggest that susceptibility to fake news is the result of lack of thinking rather than only partisan motivated reasoning (Pennycook & Rand, 2019).

Lastly, the current study also significantly advances our understanding of the theoretical mechanisms on which the intervention acts. For example, while inoculated individuals improved in their reliability assessments of the fake news items, the average confidence they expressed in their judgements also increased significantly and substantially. Importantly, the intervention only significantly increased confidence amongst those who updated their judgments in the right direction (i.e. correctly judging manipulative items to be less reliable). These findings are supported by previous literature demonstrating the certainty-bolstering effects of inoculation treatments (Tormala & Petty, 2004) and may suggest that confidence plays a key role in both prophylactic and therapeutic inoculation approaches. Yet, more research is required to identify whether an increase in confidence pertains to the fake items themselves or rather the ability to refute misinformation in general. For example, Tormala and Petty (2004) have argued that these mechanisms are likely to be intertwined as individuals might be confident in their ability to refute counterarguments because they perceive their attitudes to be valid and therefore, are both more willing and likely to defend their beliefs.

This study did suffer from a number of necessary limitations. First, we controlled for modality (given that both *Bad News* and *Tetris* are games), but lacked a condition that is cognitively comparable to the inoculation condition. It will be important for future research to evaluate to what extent “active” gamified inoculation is superior to “passive” approaches—including traditional fact-checking and other critical thinking interventions—especially in terms of eliciting a) motivation, b) the ability to help people discern reliable from fake news, and c) the rate at which the inoculation effect decays over time. Second, although we improved on the initial design by having participants evaluate simulated twitter posts (pre and post) outside of the game environment, we were not able to determine if playing the *Bad News* game led to increased ability to detect real news or changes in online behaviour (e.g. if players shared less fake news on social media than people who did not play the game). Third, the fact that a small minority of individuals appear to engage in contrary updating is worth noting and a finding future work may want to investigate further (e.g. in terms of prior motivations). Fourth, we did not examine the duration of the inoculation effect over time but we encourage future research to do so given that inoculation treatments are known to decay over time (Banas & Rains, 2010). Lastly, our *Prolific* sample was likely not representative of the U.K. population.

In conclusion, this study addressed the main shortcomings identified by Roozenbeek and van der Linden (2019) in their original evaluation of the *Bad News* game: the lack of a control group, a relatively small number of items to measure effectiveness, and the absence of attitudinal certainty measurements. We conclude that, compared to a control group, the generalized inoculation intervention not only successfully conferred resistance to online manipulation, but also boosted confidence in the ability to resist fake news and misinformation.

## Data Accessibility Statement

The raw dataset necessary to reproduce the analyses reported in this paper can be retrieved from https://figshare.com/s/818c1a38da814b0bdf20.

## Additional Files

The additional files for this article can be found as follows:

10.5334/joc.91.s1 Supplementary Table 1. Average reliability (pre-post) judgments overall and for each fake news badge by experimental condition.

10.5334/joc.91.s2 Supplementary Table 2. Average confidence (pre-post) judgments overall and for each fake news badge by experimental condition.

10.5334/joc.91.s3 Supplementary Figure 1. Mean reliability judgments by condition (pre-test).

10.5334/joc.91.s4 Supplementary Figure 2. Mean reliability judgments by condition (post-test).

10.5334/joc.91.s5 Supplementary Figure 3. Mean confidence judgments by condition (pre-test).

10.5334/joc.91.s6 Supplementary Figure 4. Mean confidence judgments by condition (post-test).

10.5334/joc.91.s7 Supplementary Figure 5. All 18 fake news items participants viewed pre-post by badge.

10.5334/joc.91.s8 Supplementary Figure 6. Scree plot for reliability judgments following PCA.

10.5334/joc.91.s9 Supplementary Figure 7. Scree plot for confidence judgments following PCA.

## Footnotes

In the original study by Roozenbeek and van der Linden (2019), only six items were included. We included the original items plus two new ones for each badge using the same approach.

Socio-demographics (except for ideology) were answered by 52% (*n* = 104) of the 197 participants.

A linear regression with post-test as the dependent variable, condition as a dummy, and pre-test as a covariate gives the same result. There was no significant difference at pre-test between the conditions (*M*_inoculation_ = 3.14 vs. *M*_control_ = 3.32, *M*_diff_ = –0.185 95% CI [–0.42 0.05], *p* = 0.12, see Supplementary Table S1 and Figs S1–2).

Though conservatives (*M* = 3.56) were significantly more susceptible than liberals (*M* = 3.05) on the pre-test, *t*(147) = 3.22, *d* = 0.61, *p* < 0.01, consistent with Roozenbeek and van der Linden (2019).

A linear regression with post-test as the dependent variable, condition as a dummy, and pre-test as a covariate gives the same result. There was no significant difference in confidence judgments at pre-test between conditions (*M*_inoculation_ = 5.25 vs. *M*_control_ = 5.27, *M*_diff_ = 0.02 95% CI [–0.24 0.20], *p* = 0.88, please see Supplementary Table S2 and Figures S3–4).

Meaning that fake headlines were deemed less reliable on the post-test compared to the pre-test (i.e. *M*_diff_ < 0).
