# Supplementary material for: Good News about Bad News: Gamified Inoculation Boosts Confidence and Cognitive Immunity Against Fake News
Source: J Cogn. 2020 Jan 10;3(1):2. doi: 10.5334/joc.91 (PMC6952868; doi:10.5334/joc.91)
Supplement: Supplementary Table 1. — Average reliability (pre-post) judgments overall and for each fake news badge by experimental condition. [file joc-3-1-91-s1.pdf]

Supplementary Information

	Experimental condition								
	Inoculation (n = 96)				Control (n = 102)				
	M_{pre}	M_{post}	M_{diff}	$95\%CI_{diff}$	M_{pre}	M_{post}	M_{diff}	$95\%CI_{diff}$	Cohen's d
**Fake news scale**	3.14	2.69	-0.45	[-0.29, -0.61]	3.32	3.23	-0.09	[-0.03, -0.15]	0.60
**Impersonation**	3.22	2.76	-0.46	[-0.21, -0.70]	3.49	3.48	-0.01	[-0.16, 0.14]	0.45
**Polarisation**	2.85	2.59	-0.26	[-0.03, -0.48]	3.07	2.95	-0.12	[-0.27, 0.02]	0.14
**Conspiracy**	3.13	2.58	-0.55	[-0.33, -0.77]	3.47	3.27	-0.20	[-0.04, -0.36]	0.36
**Emotion**	3.39	2.87	-0.52	[-0.29, -0.74]	3.53	3.44	-0.09	[-0.23, 0.05]	0.45
**Discrediting**	3.36	2.80	-0.56	[-0.33, -0.79]	3.39	3.41	0.02	[-0.19, 0.14]	0.58
**Trolling**	2.83	2.52	-0.31	[-0.12, -0.49]	2.96	2.83	-0.13	[-0.02, 0.28]	0.22

Supplementary Table 1. Average reliability (pre-post) judgments overall and for each fake news badge by experimental condition.
