# Supplementary material for: Good News about Bad News: Gamified Inoculation Boosts Confidence and Cognitive Immunity Against Fake News
Source: J Cogn. 2020 Jan 10;3(1):2. doi: 10.5334/joc.91 (PMC6952868; doi:10.5334/joc.91)
Supplement: Supplementary Table 2. — Average confidence (pre-post) judgments overall and for each fake news badge by experimental condition. [file joc-3-1-91-s2.pdf]

	Experimental condition								
	**Inoculation** (n = 96)				**Control** (n= 102)				
	M_{pre}	M_{post}	M_{diff}	95%CI_{diff}	M_{pre}	M_{post}	M_{diff}	95% CI_{diff}	Cohen's *d*
**Fake news scale**	5.25	5.47	0.22	[0.10, 0.34]	5.27	5.21	-0.06	[-0.03, 0.14]	0.52
**Impersonation**	5.56	5.71	0.15	[-0.05, 0.35]	5.68	5.51	-0.17	[-0.00, 0.34]	0.34
**Polarisation**	5.13	5.34	0.21	[0.01, 0.41]	5.11	5.10	-0.01	[-0.14, 0.16]	0.25
**Conspiracy**	5.14	5.40	0.26	[0.08, 0.44]	5.10	5.12	0.02	[-0.20, 0.15]	0.27
**Emotion**	5.15	5.41	0.26	[0.10, 0.42]	5.25	5.12	-0.13	[-0.03, 0.29]	0.49
**Discrediting**	5.28	5.46	0.18	[0.01, 0.35]	5.19	5.17	-0.02	[-0.15, 0.20]	0.23
**Trolling**	5.24	5.48	0.24	[0.05, 0.42]	5.26	5.24	-0.02	[-0.13, 0.17]	0.31

Supplementary Table 2. Average confidence (pre-post) judgments overall and for each fake news badge by experimental condition.
