# Supplementary figures and images for: Good News about Bad News: Gamified Inoculation Boosts Confidence and Cognitive Immunity Against Fake News
Source: J Cogn. 2020 Jan 10;3(1):2. doi: 10.5334/joc.91 (PMC6952868; doi:10.5334/joc.91)

*Supplementary Figure 1. Mean reliability judgments by condition (pre-test)*

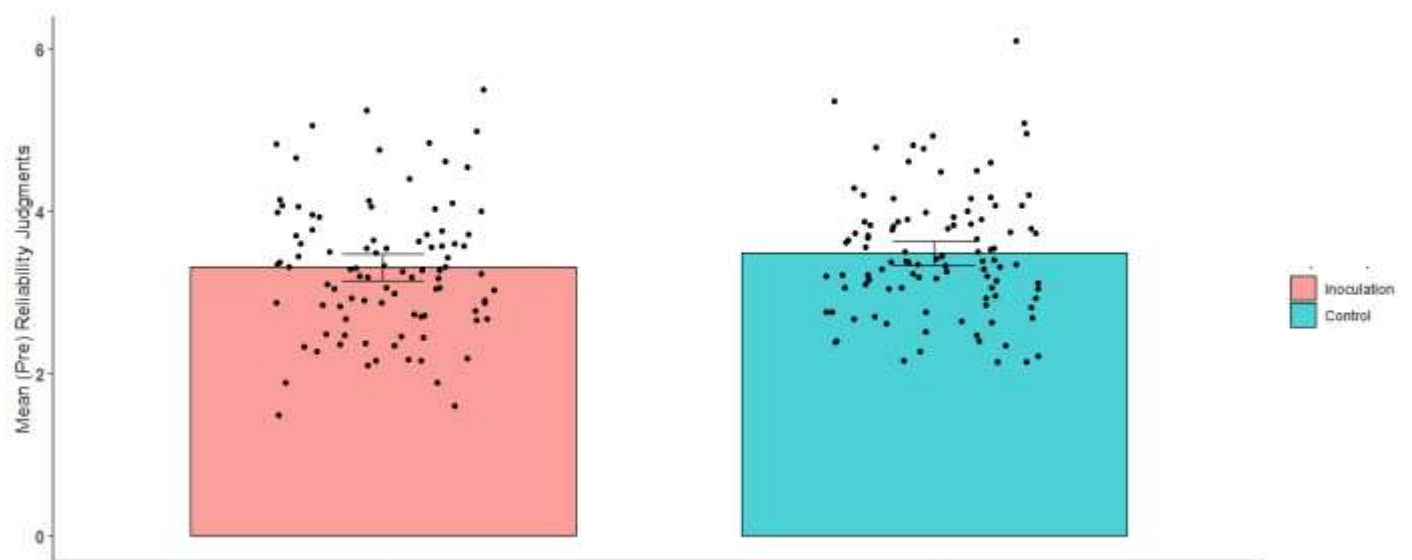

Supplement: Supplementary Figure 1. — Mean reliability judgments by condition (pre-test). [file joc-3-1-91-s3.pdf]

*Supplementary Figure 2. Mean reliability judgments by condition (post-test)*

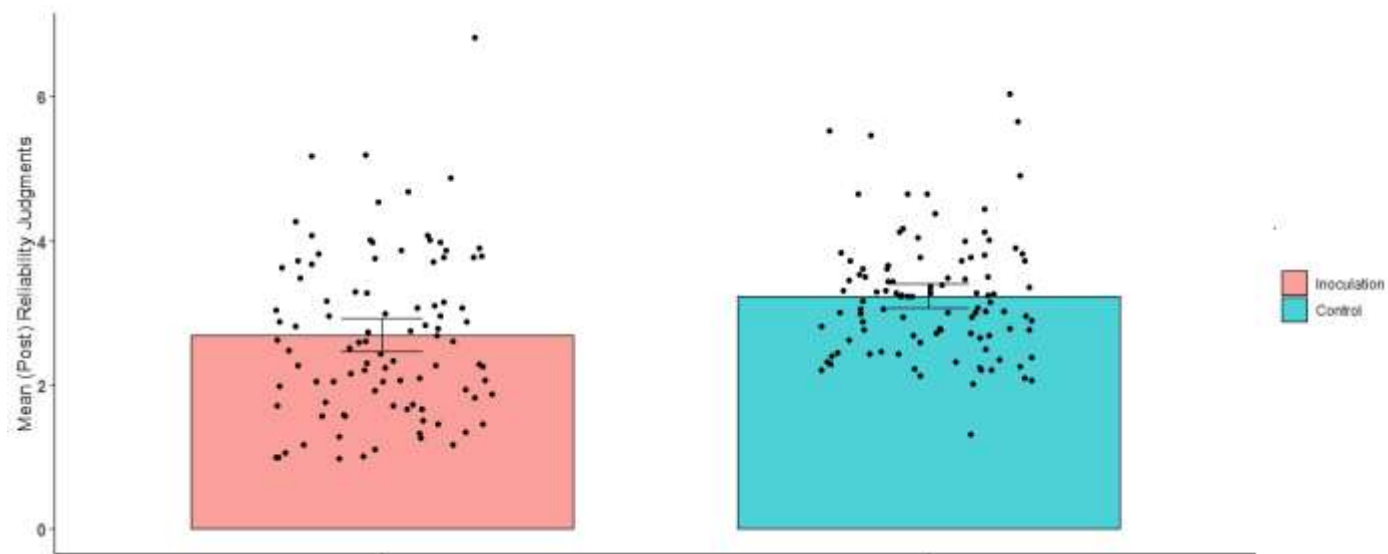

Supplement: Supplementary Figure 2. — Mean reliability judgments by condition (post-test). [file joc-3-1-91-s4.pdf]

Supplementary Figure 3. Mean confidence judgments by condition (pre-test)

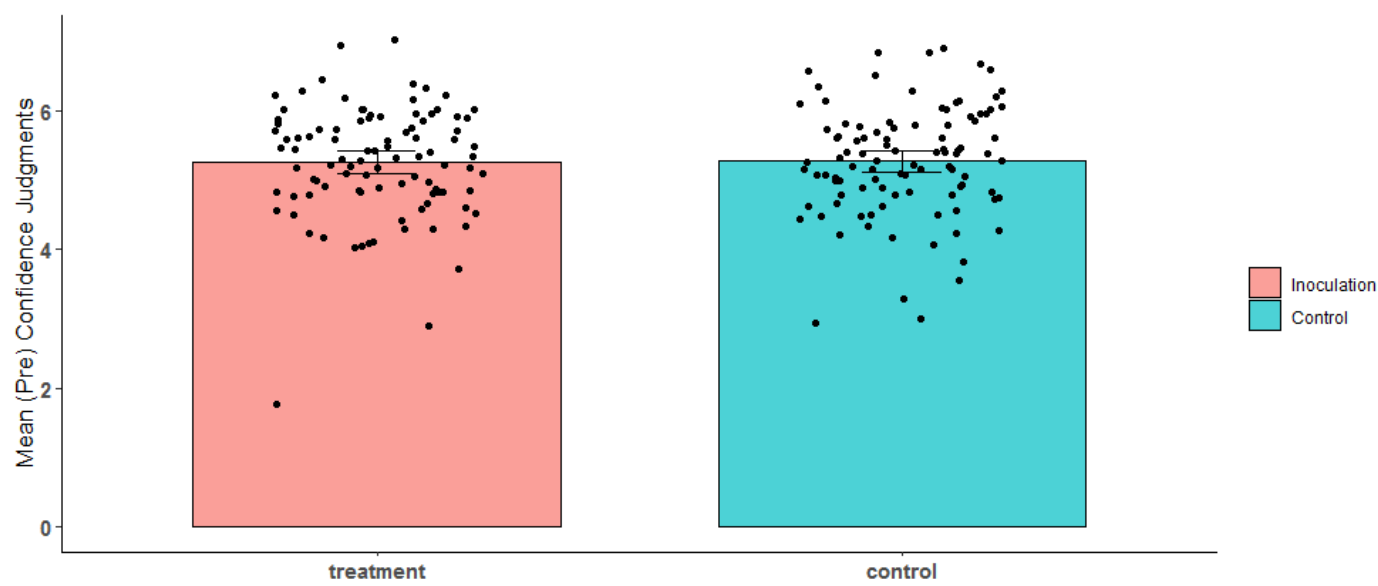

Supplement: Supplementary Figure 3. — Mean confidence judgments by condition (pre-test). [file joc-3-1-91-s5.pdf]

Supplementary Figure 4. Mean confidence judgments by condition (post-test)

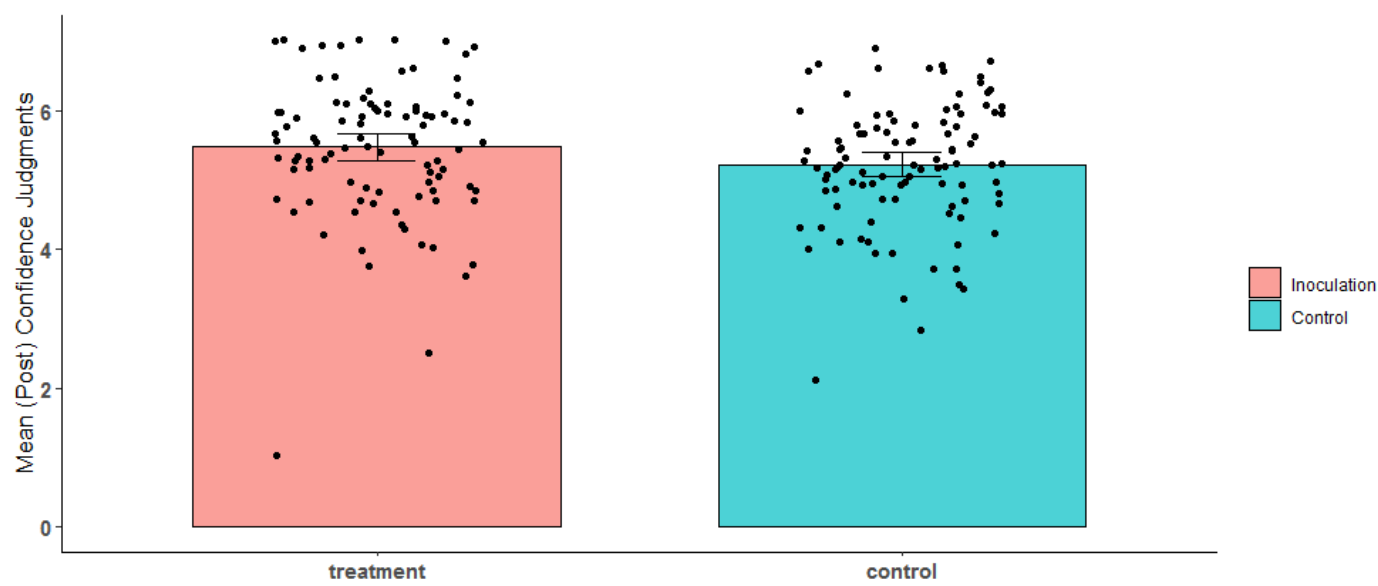

Supplement: Supplementary Figure 4. — Mean confidence judgments by condition (post-test). [file joc-3-1-91-s6.pdf]

Supplementary Figure 6. Scree plot for reliability judgments following PCA.

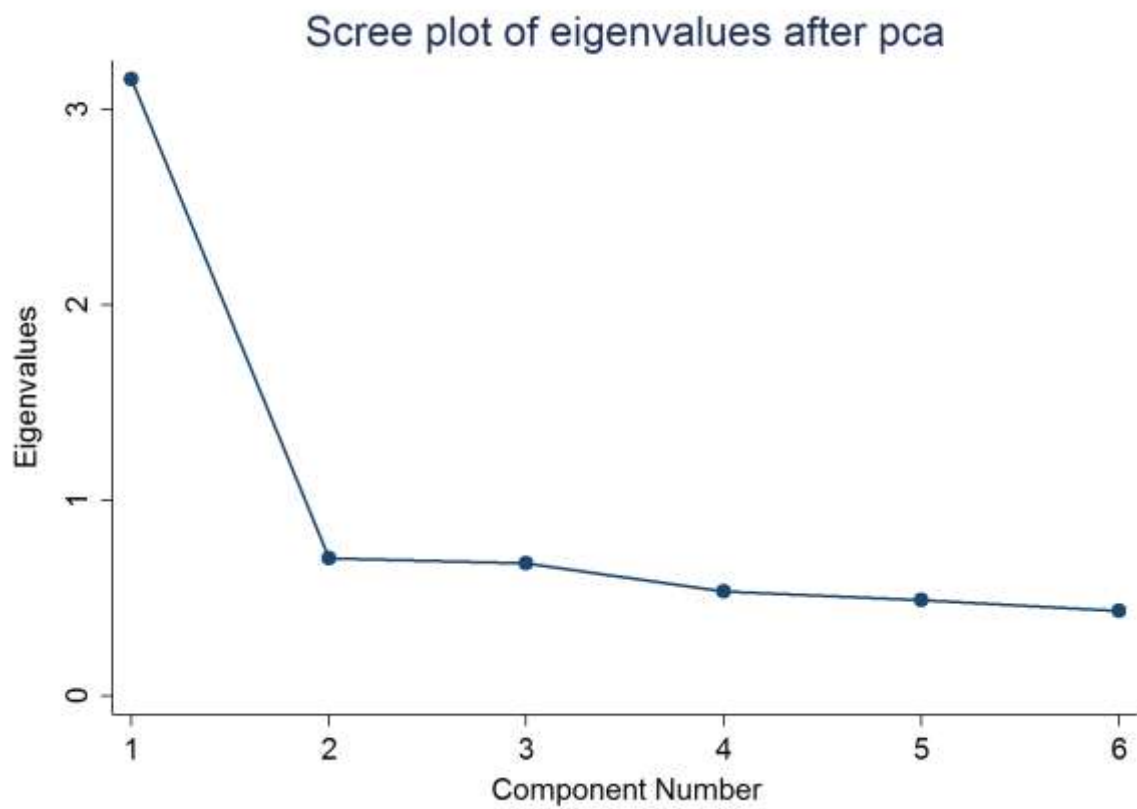

Supplement: Supplementary Figure 6. — Scree plot for reliability judgments following PCA. [file joc-3-1-91-s8.pdf]

Supplementary Figure 7. Scree plot for confidence judgments following PCA.

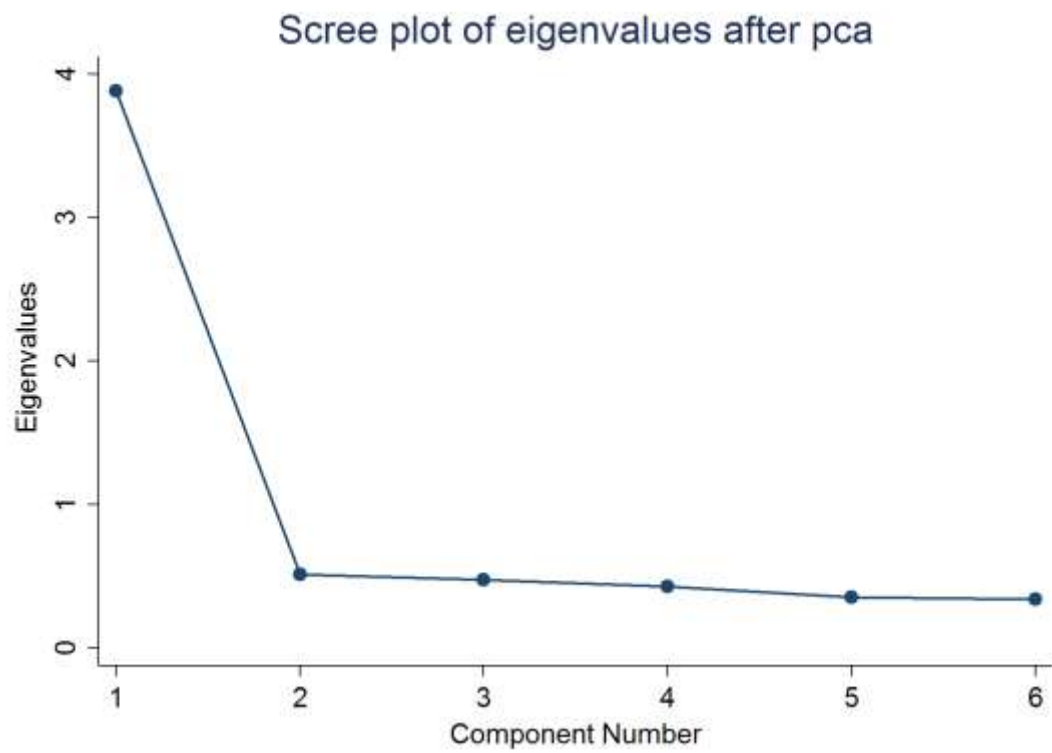

Supplement: Supplementary Figure 7. — Scree plot for confidence judgments following PCA. [file joc-3-1-91-s9.pdf]
