# Supplementary material for: Good News about Bad News: Gamified Inoculation Boosts Confidence and Cognitive Immunity Against Fake News
Source: J Cogn. 2020 Jan 10;3(1):2. doi: 10.5334/joc.91 (PMC6952868; doi:10.5334/joc.91)
Supplement: Supplementary Figure 5. — All 18 fake news items participants viewed pre-post by badge. [file joc-3-1-91-s7.pdf]

Supplementary Figure 5. All 18 fake news items participants viewed pre-post by badge

Impersonation

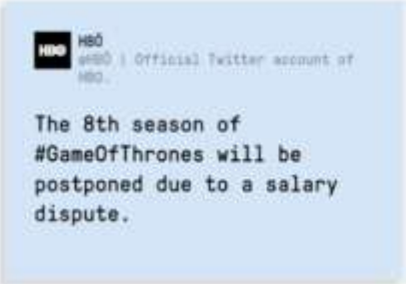

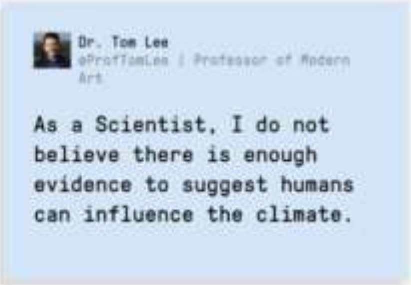

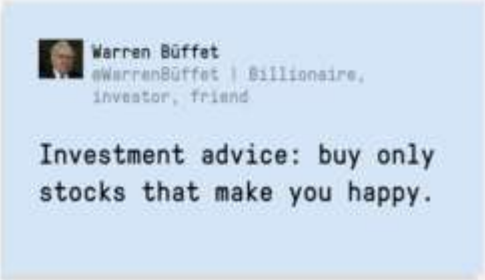

Emotional Content

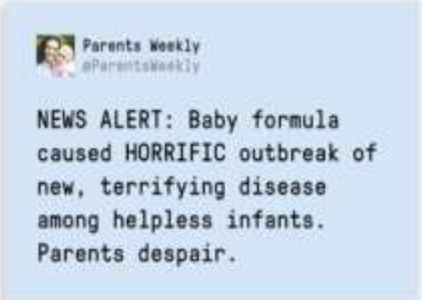

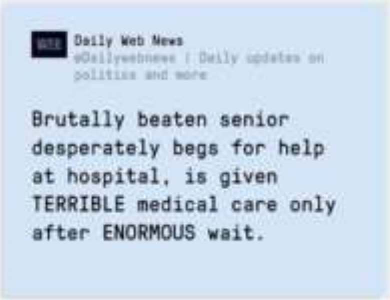

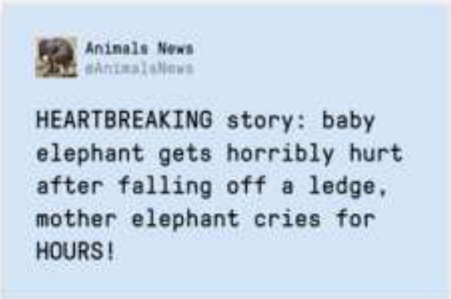

Conspiracy Theories

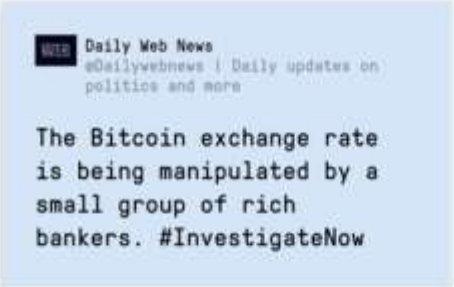

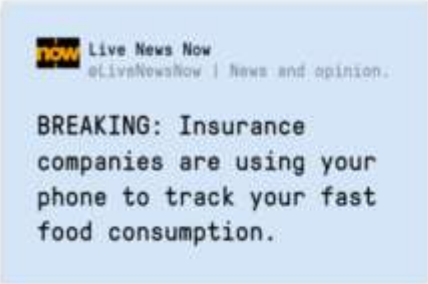

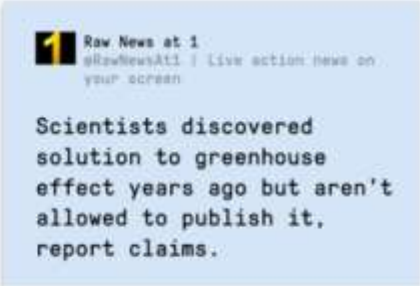

Discrediting Opponents

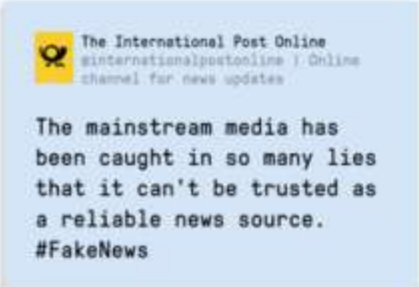

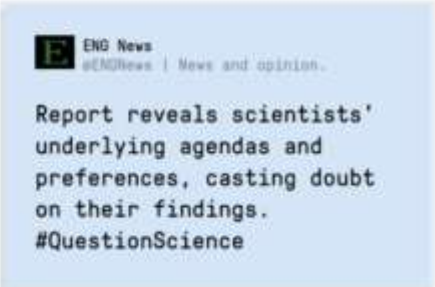

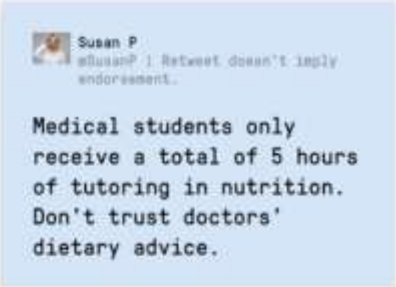

Polarisation

The Daily Chronicle @DailyChron

New study shows right-wing people lie much more than left-wing people.

Next Global @NextGlobal

Worldwide rise of left-wing extremist groups damaging world economy: UN report.

Rapid Updates @RapidUpdates

The myth of "equal IQ" between left-wing and right-wing people exposed. #TruthMatters

Trolling

Quad Media @QuadMedia

Another shark loan for developing countries @WorldBank? #WorldOfExtortion #HumanBanking

Johnson Crude Oil @JohnsonCrudeOil

Hey @LeoDiCaprio, it's snowing and freezing in New York. Could use some of that global warming you're always going on about!

Joe Stephensohn @NoSchmoeJoe

A sandwich maker at Subway just took a bite out of my sub! Are you starving your employees @Subway?
